# Five-year longitudinal changes in thigh muscle mass of septuagenarian men and women assessed with DXA and MRI

**DOI:** 10.1007/s40520-019-01248-w

**Published:** 2019-08-02

**Authors:** James Cameron, Jamie S. McPhee, David A. Jones, Hans Degens

**Affiliations:** 1 Department of Health Professions, Manchester Metropolitan University, Manchester, UK; 2 Department of Sport and Exercise Sciences, Manchester Metropolitan University, Manchester, UK; 3 Department of Life Sciences, Faculty of Science and Engineering, Manchester Metropolitan University, John Dalton Building, Manchester, M15GD UK; 4 Institute of Sport Science and Innovations, Lithuanian Sports University, Kaunas, Lithuania; 5 Medicine and Pharmacy, Targu Mures University, Targu Mures, Romania

**Keywords:** Muscles, Ageing, Body composition, Validity, MRI, DXA

## Abstract

Magnetic resonance imaging (MRI) and dual-energy X-ray absorptiometry (DXA) were used to assess changes in thigh lean mass in septuagenarian men and women during a 5-year longitudinal study. Twenty-four older individuals participated in the study (10 men: 71.6 ± 4.1 years; 14 women: 71.3 ± 3.2 years at baseline). Thigh MRI and whole-body DXA scans were used to estimate changes in thigh lean mass. Both MRI and DXA showed that thigh lean mass was reduced by approximately 5% (*P* = 0.001) over the 5-year period in both men and women. The percentage loss of muscle mass determined with MRI and DXA showed moderate correlation (*R*^2^ = 0.466; *P* < 0.001). Bland–Altman analysis showed that the average change over 5 years of follow-up measured by DXA was only 0.18% greater than MRI, where the limits of agreement between DXA and MRI were ± 10.4%. Baseline thigh lean mass did not predict the percentage loss of thigh lean mass over the 5-year period (*R*^2^ = 0.003; *P* = 0.397), but a higher baseline body fat percentage was associated with a larger loss of thigh muscle mass in men (*R*^2^ = 0.677; *P* < 0.003) but not in women (*R*^2^ = 0.073; *P* < 0.176). In conclusion, (1) DXA and MRI showed a similar percentage loss of muscle mass over a 5-year period in septuagenarian men and women that (2) was independent of baseline muscle mass, but (3) increased with higher baseline body fat percentage in men.

## Introduction

Ageing is accompanied by changes in muscle mass that are thought to contribute to reduced physical function and vigour, and the eventual loss of independence in old age [1]. This loss of muscle mass and physical function has been described as sarcopenia [2]. By the eighth decade, muscle mass has declined by around 30% from peak values, with these losses principally coming from the atrophy of type II fibres [3] and loss of muscle fibres [4]. The loss of myofibers is thought to be a consequence of motor neuron death and it has been reported that up to 50% of motor units are lost by the eighth decade [5].

The major problem with most studies of human ageing is that they are cross sectional, and it is important to develop and validate methods to assess changes in muscle mass in longitudinal studies. Muscle imaging techniques allow the non-invasive evaluation of skeletal muscle size and architecture [6] and include computer axial tomography (CT), dual energy X-ray absorptiometry (DXA), and magnetic resonance imaging (MRI) [7–10]. CT and MRI are generally considered the gold standard, allowing the accurate assessment of muscle cross-sectional area, muscle mass and intramuscular adipose tissue area. Nonetheless, these techniques are expensive and consequently DXA [11] and bioelectrical impedance [12] are frequently used to identify sarcopenia.

Previously, good correlations have been found between muscle size estimations with CT, DXA and MRI in similar groups of individuals [9, 13–16]. Despite a strong correlation (*R*^2^ = 0.90 young, *R*^2^ = 0.83 old) in a large cross-sectional cohort study, thigh muscle mass was overestimated by DXA as the slope of the DXA–MRI relationship was steeper than 1 and had an intercept of approximately 0.4 kg [15]. In addition, DXA underestimated the percentage difference in muscle mass between young adults and older people [15], suggesting that DXA underestimates the age-related loss of muscle mass. However, comparison of longitudinal studies that either use DXA or CT have shown similar losses of thigh muscle mass in older people [17–19]. It remains to be seen, whether longitudinal changes in skeletal muscle mass of older people determined with DXA are indeed similar to that seen with MRI when both methods are applied to the same individuals, or that this method underestimates the loss of muscle mass beyond the age of 70 years.

Therefore, the purpose of the present study was to compare changes in muscle mass as measured by DXA and MRI in a 5-year longitudinal study of men and women in their eighth decade. It was hypothesised that DXA underestimates the percentage loss of muscle mass when compared with MRI. In addition, we studied whether the rate of muscle loss is (1) negatively related to baseline muscle mass and/or (2) positively related to baseline body fatness.

## Methods

### Participants and ethical approval

The participants are a subgroup from the cross-sectional MYOAGE study (http://www.myoage.eu) [20]. The participants were recruited from local groups such as the University of the Third Age, further learning, history, teaching children, church organisations or arts and crafts (Manchester, UK) and were asked 5 years later to return for a follow-up study. Data presented in this report are from the 24 older participants that returned (10 men, 14 women). The original study contained 25 men and 28 women at baseline, and their MRI lean muscle mass did not differ significantly from the group that returned after 5 years, indicating that our sub population is representative of the baseline cohort. Written informed consent was obtained from each participant before partaking in both the first and the follow-up study. The studies conformed to the Declaration of Helsinki and were approved by the local ethics committee of the Manchester Metropolitan University. Participant characteristics are presented in Table 1. All individuals were community dwelling, socially active and classed as healthy. Exclusion criteria were: known musculoskeletal or cardiovascular diseases, any limb fractures within 5 years of testing, hip or knee replacement in the previous 2 years, immobilised for greater than 1 week 3 months prior to testing, institutionalisation, unable to walk 250 m unassisted, chronic pain syndrome, metabolic disease, chronic obstructive pulmonary disease, or neurological disorders (e.g. Parkinson’s).

**Table 1:** Participant characteristics

	Women (*n* = 14)			Men (*n* = 10)			Statistical comparisons		
	Baseline	Follow-up	% Change	Baseline	Follow-up	% Change	Time	Gender	Gender × Time
Age (years)	71.3 ± 3.2	76.2 ± 3.3		71.6 ± 4.1	76.2 ± 4.4		***P* = 0.000**	*P* = 0.923	*P* = 0.193
Body mass (kg)	65.5 ± 10.4	63.4 ± 10.9	− 3.5	83.6 ± 15.2	83.9 ± 15.1	0.5	*P* = 0.079	***P* = 0.000**	***P* = 0.029**
Height (m)	1.61 ± 0.07	1.60 ± 0.06	− 0.5	1.74 ± 0.08	1.73 ± 0.08	− 0.5	***P* = 0.000**	***P* = 0.000**	*P* = 0.874
BMI (kg/m^2^)	25.6 ± 5.47	25.0 ± 5.5	− 2.0	27.7 ± 4.4	28.1 ± 4.1	1.5	*P* = 0.798	*P* = 0.123	***P* = 0.019**
FFM (kg)	39.0 ± 3.1	37.7 ± 3.1	− 3.5	55.3 ± 8.1	54.5 ± 7.5	− 1.5	***P* = 0.001**	***P* = 0.000**	*P* = 0.378
FM (kg)	24.1 ± 9.4	23.5 ± 10.3	2.5	25.0 ± 10.4	26.1 ± 10.3	4.5	*P* = 0.580	*P* = 0.604	*P* = 0.075
FM (%)	37.0 ± 9.2	36.9 ± 9.94	0	30.2 ± 9.2	31.5 ± 8.7	4.5	*P* = 0.236	*P* = 0.056	*P* = 0.172
ALM (kg)	17.4 ± 1.8	16.7 ± 1.75	− 4.0	25.7 ± 4.0	24.6 ± 3.7	− 5	***P* = 0.000**	***P* = 0.000**	*P* = 0.206
BMD (g/mm^2^)	1.07 ± 0.10	1.07 ± 0.10	0	1.25 ± 0.12	1.26 ± 0.12	1.0	*P* = 0.352	***P* = 0.000**	*P* = 0.519

Significant values are in bold

Data shown as mean ± SD

*BMI* body mass index, *FFM* fat-free mass, *FM* fat mass, *ALM* appendicular lean mass, *BMD* bone mineral density

### Anthropometry

While wearing light indoor clothing, body mass was recorded on a digital scale to the nearest 0.1 kg. Standing height was measured using a stadiometer to the nearest 1 mm. Body mass index (BMI) was calculated as body mass (kg)/(height (m)^2^).

### Dual-energy X-ray absorptiometry (DXA)

**Fig. 1:**
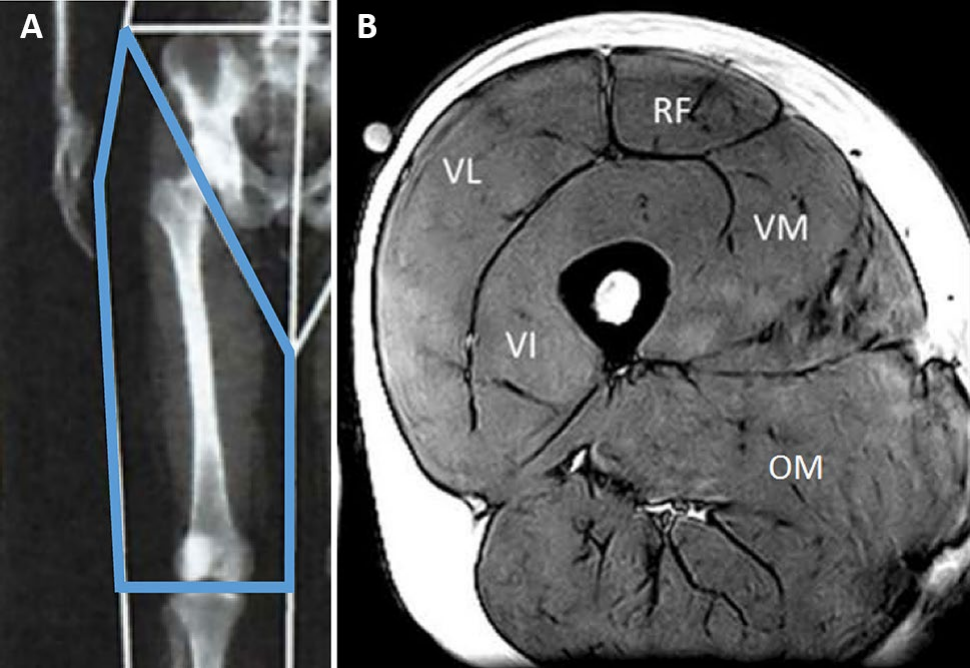
**a** Example of dual-energy X-ray absorptiometry (DXA) image showing regions of interest of the thigh. **b** Magnetic resonance imaging (MRI) image of the thigh muscles. *VI* vastus intermedius, *VL* vastus lateralis, *VM* vastus medialis, *RF* rectus femoris, *OM* other muscles

Participants lay supine on the scanning bed wearing a medical gown. A total body DXA (Lunar Prodigy Advance, GE Healthcare, Chicago, USA) scan was performed to measure total body composition and bone mineral density. Estimations of total lean mass and fat mass were obtained using Prodigy, Encore 2006 v10.50.086 software (GE Healthcare). To estimate the fat mass, bone mineral content and lean mass in the thigh of the dominant leg, the thigh was demarcated by one border proximally and parallel to the greater trochanter and another through the knee joint line, as described previously [15, 16] (Fig. 1a). All DXA analyses were completed by the same investigator. Each standard total body scan took 295 s with an estimated skin entrance dose of 0.4 µGy (GE Healthcare, Lunar encore, Safety and Specification Manual). Typically, the estimates of lean mass by DXA software packages include connective tissue, non-mineral components of bone and non-adipose components of fat tissue alongside muscle mass. As the contribution of these factors is unclear and possible changes of these components with ageing are unknown, we did not correct for these potential confounders. The system was calibrated with the same whole-body phantom at baseline and at 5-year follow-up before each scan.

### Magnetic resonance imaging (MRI)

In six of the participants, thigh volume was measured using a 0.25-T MRI scanner (G-Scan, Esaote, Genova, Italy). The participant was in a supine position in the scanner and multiple 3.1-mm-thick serial transverse sections were acquired every 25 mm from the proximal to the distal heads of the femur of the dominant leg using a turbo 3D T1-weighted protocol (matrix 256 × 256, TR 40 ms, TE 16 ms). The cross-sectional area of the four quadriceps muscles and other thigh muscles (hamstrings, abductors and adductors) in each slice (Fig. 1b) were determined using computer imaging software (OsiriX medical imaging software, OsiriX, Atlanta, USA). We have previously shown that thigh muscle volume can be calculated from a single scan [21, 22]. In a subset of six participants, we found a good correlation between the measured and the calculated thigh muscle volume (*R*^2^ = 0.89; *P* = 0.007) in this cohort. Consequently, in the remaining 18 participants, thigh volume was estimated from a single scan taken at 60% of the length from distal-to-proximal femur [22].

### Statistics

Data were analysed using SPSS v22 (IBM, 2015). A Shapiro–Wilk test showed that the data were normally distributed. Repeated measures ANOVA with “within subject factor” time (baseline and follow-up) and “between subject factor” gender was used. A gender–time interaction indicated that men and women changed differently over time. Linear regression analysis was conducted to consider correlations between measurements. Statistical significance was accepted as *P* < 0.05. Data were expressed as mean ± standard deviation unless stated otherwise. Bland–Altman analysis [23] was used to determine the limits of agreement between DXA and MRI. Bland–Altman plots illustrate the agreement between two measurement techniques. The test–retest variability was given as the coefficient of variation (CVp), which was calculated as the SD of the differences between MRI and DXA as a proportion of the mean: CVp = √((∑CVi^2^)/*n*).

## Results

### Participant characteristics

Women were shorter, had lower FFM, ALM and BMD than men (Table 1; *P* < 0.001). The participants lost about 1 cm in stature over the 5-year period as well as having lower FFM and ALM (*P* ≤ 0.001), irrespective of gender. The gender–time interactions for body mass (*P* = 0.029) and BMI (*P* = 0.019) were reflected by a decrease in body mass and BMI in women, but not in men. There was no significant change in fat mass and BMD over the 5-year period.

### Correlations between MRI and DXA

**Fig. 2:**
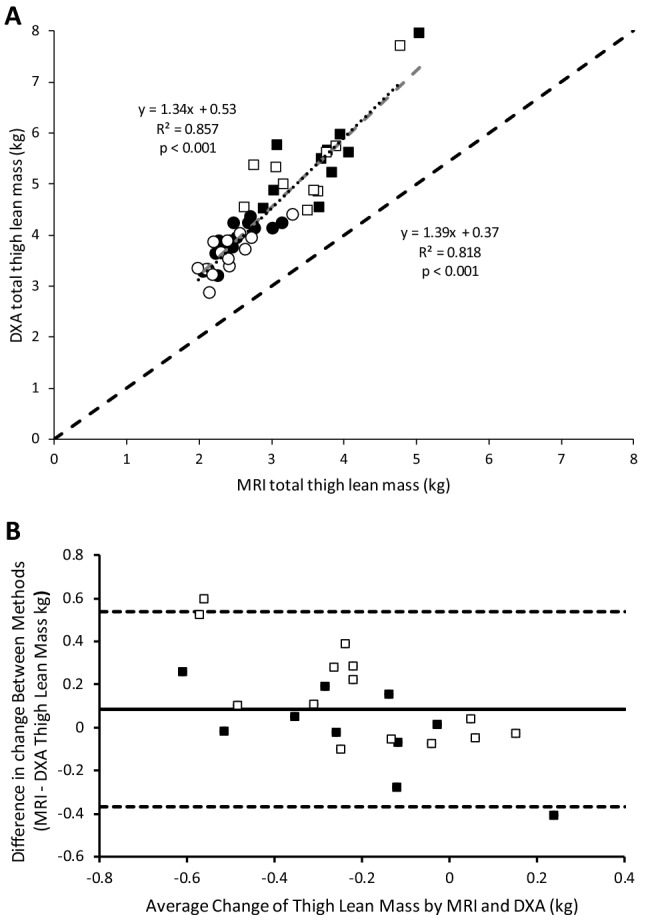
**a** The relationship between thigh lean mass as estimated by DXA vs. that estimated by MRI. ■: men and ●: women at baseline, and □: men and ○: women at follow-up.  : line of identity;  : regression line at baseline; ···: regression line at follow-up. Equations—left: baseline; right: follow-up. **b** Bland–Altman plot to show the absolute agreement between MRI and DXA; ■: men and □: women. Horizontal dashed lines represent 1.96 standard deviation above and below the average difference between methods, depicting levels of agreement (+ 0.54 kg upper level of agreement and − 0.37 kg lower level of agreement). Solid horizontal line represents the bias between methods (DXA shows a 0.09 kg larger loss of muscle mass than MRI over the 5-year period)

Both MRI and DXA showed that men had larger muscles than women (Table 2; *P* ≤ 0.001). Figure 2a shows the correlation between thigh muscle size as measured by DXA and MRI for values at baseline (*R*^2^ = 0.857; *P* < 0.001) and at the 5-year follow-up (follow-up *R*^2^ = 0.818; *P* < 0.001). The regression lines of the two correlations were similar.

**Table 2:** Measurements of thigh muscle size by dual-energy X-ray absorptiometry (DXA) and magnetic resonance imaging (MRI)

	Women (*n*=14)			Men (*n*=10)			Significant differences		
	Baseline	Follow-up	% Change	Baseline	Follow-up	% Change	Time	Gender	Gender × time
MRI quadriceps muscle lean mass (kg)	1.05 ± 0.16	1.01 ± 0.16	− 4.5	1.63 ± 0.31	1.48 ± 0.28	− 8.8	***P *= 0.001**	***P *= 0.001**	*P***= **0.61
MRI other muscle lean mass (kg)	1.39 ± 0.15	1.30 ± 0.18	− 6.4	1.94 ± 0.34	1.86 ± 0.35	− 3.8	***P *= 0.001**	***P *= 0.001**	*P***= **0.756
MRI total thigh lean mass (kg)	2.44 ± 0.29	2.31 ± 0.31	− 5.5	3.56 ± 0.57	3.35 ± 0.57	− 6.1	***P *= 0.001**	***P *= 0.001**	*P***= **0.228
DXA thigh lean mass (kg)	3.89 ± 0.36	3.59 ± 0.40	− 8.0	5.55 ± 0.98	5.34 ± 0.93	− 4.0	***P *= 0.001**	***P *= 0.001**	*P***= **0.529

Significant values are in bold

**Fig. 3:**
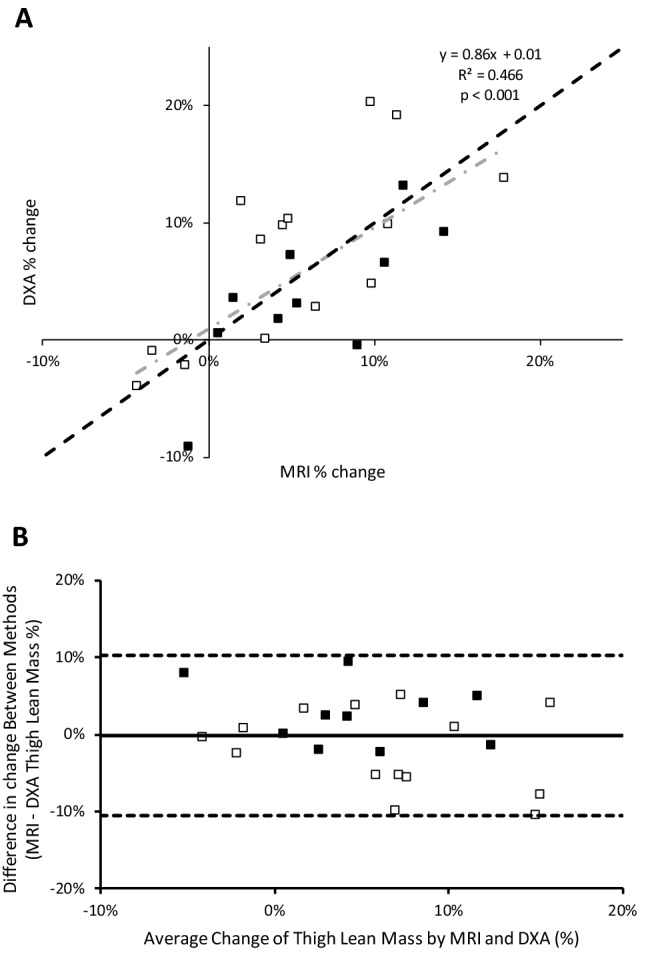
**a** The relationship in men and women between thigh lean mass percentage change as estimated by DXA vs. MRI. ■: men and □: women;  : line of identity;  : regression. **b** Bland–Altman plot to show the percentage agreement between MRI and DXA. ■: men and □: women. Horizontal dashed lines represent 1.96 standard deviation above and below the average difference between methods, depicting levels of agreement (+ 10.2% upper level of agreement and − 10.6% lower level of agreement). Solid horizontal line signifies the 0.18% larger decrease in muscle size determined by DXA than by MRI

When analysing data showing the changes to muscle size over the 5-year follow-up, Bland–Altman plots (Fig. 2b) showed a 0.09 kg larger loss measured by DXA compared with that measured by MRI. Limits of agreement between DXA and MRI was ± 0.453 kg. The percentage loss of muscle mass determined with MRI and DXA showed moderate correlation (*R*^2^ = 0.466; *P* < 0.001; Fig. 3a). Bland–Altman plots (Fig. 3b) show a 0.18% lower muscle loss measured by MRI compared with DXA and the limits of agreement between DXA and MRI was ± 10.4%. The overall pooled co-efficient of variation (pCV) between MRI and DXA over 5 years was 0.045%.

### Longitudinal changes in thigh lean mass

MRI showed a similar percentage decrease in thigh muscle size in men and women (Table 2). The percentage loss of thigh lean mass did not differ significantly between DXA and MRI (*P* = 0.841), as indicated by similar MRI:DXA ratios for thigh muscle size at baseline and 5-year follow-up (*P* = 0.967).

It is possible to distinguish the quadriceps and other muscles in the thigh with MRI to investigate possible differential atrophy between thigh muscles. The ratio of quadriceps to other muscles was similar in both genders at baseline (Table 2; *P* = 0.224) and the absence of a significant age-related change in this ratio (Table 2; *P* = 0.517) indicated that the atrophy was similar in both muscle compartments.

There was no significant correlation between baseline thigh muscle volume and the percentage decline over the 5-year period in either men or women when measured by either MRI (*R*^2^ = 0.003; *P* = 0.397) or DXA (*R*^2^ = 0.009; *P* = 0.326). However, baseline body fat percentage was associated with a larger percentage decrease in muscle volume following the 5-year period in men (MRI: *R*^2^ = 0.677; *P* = 0.003; DXA: *R*^2^ = 0.308; *P* = 0.048), but not in women (MRI: *R*^2^ = 0.073; *P* = 0.176; DXA: *R*^2^ = 0.024; *P* = 0.298).

## Discussion

We have previously shown that, when comparing young and old, MRI measurements suggest a greater age-related decline in muscle mass than that obtained from DXA measurements. DXA is a convenient method to assess body composition and muscle mass but our previous cross-sectional observations raised concerns about its suitability for use in longitudinal studies of changes in muscle mass and progression of sarcopenia. In the present study we have extended this to show that the percentage loss of muscle mass over a 5-year period was similar for DXA and MRI. We also showed that the percentage loss of muscle mass in the 5-year period was similar in (1) quadriceps and hamstring muscles, (2) recreationally active healthy older men and women, (3) was independent of baseline muscle mass, (4) was greater in men with a higher baseline body fat percentage. The estimated rate of decline in muscle mass was similar to that seen in previous longitudinal studies [17–19] and was higher than that estimated from cross-sectional comparisons of healthy recreationally active people aged in their 20 s compared with those in their 70 s.

### DXA vs. MRI

MRI total volume measurements are widely regarded as the gold standard, though these measurements are both time consuming and costly. The cost and time can be reduced significantly by calculating muscle volume from a single MRI scan. Here, we support the literature suggesting that this is not only possible in young [22] and older adults [24] and add the finding that the percentage decrease in thigh muscle cross-sectional area over a 5-year period correlated strongly with the percentage decrease in thigh muscle volume (*y* = 1.03*x*–0.04; *R*^2^ = 0.875; *P* = 0.003). This indicates that estimating changes in thigh muscle size from single MRI transverse sections taken at 60% of femur length are sufficient to assess changes in thigh muscle volume. This is likely due to the fact that muscle origins and insertions did not change over time, so any change to muscle cross-sectional area is proportional to the change in muscle volume.

While the use of a single MRI scan already saves considerable time, and hence cost, MRI and CT are not commonly available. DXA has become a popular modality to assess body composition and muscle mass in large cohort studies, due to its wider availability and ease of use [25–29]. Although we found a good correlation between the thigh muscle mass determined by DXA and MRI in both men and women at both baseline and follow-up, DXA consistently overestimated the muscle mass due to a positive intercept and a slope of the regression line greater than 1. Such a positive intercept has been seen before in young adults and older people [9, 14, 30]. This is also in line with previous work reporting a positive intercept and a slope steeper than 1 [15]. It has been suggested that protein or other material in adipose tissue may contribute to this over estimation of muscle mass by DXA [31, 32]. However, adjustments to account for connective tissue, fat infiltration and none bone mineral content of bones [7, 33] did not remove this bias.

In practice, and when following changes over a relatively short time scale of 5 years, the difference between the two methods (MRI and DXA) is small. Bland–Altman analysis showed a discrepancy between the change in muscle mass as determined by MRI and DXA in absolute terms of 0.09 kg and in percentage terms of 0.18% over the 5-year period. This suggests that DXA is an acceptable method for longitudinal tracking of muscle mass in older people.

### Longitudinal age-related decline in muscle mass in older people

Ageing is associated with an overall reduction in skeletal muscle mass that contributes significantly to the loss of muscle strength [4]. This loss of strength and concomitant slowing of the muscle [34] result in an age-related reduction in muscle power that is associated with a reduced performance in the timed-up-and-go and 6-min-walking test [35]. As the proportion of older people is rising in the western world, it is important to understand sarcopenia and its progression towards frailty in the older person [36]. Here, we found with both DXA and MRI that over the relatively short period of 5 years, muscle mass decreases by ~ 5% in people in their 70s. This is relatively more than the 25% lower muscle mass seen in a cross-sectional comparison of recreationally active people in their 70s and their 50 years younger counterparts in their 20s [24]. It is, however, similar to that seen in previous longitudinal studies in older people of a similar age as our population [17–19]. It indicates that the age-related rate of muscle decline is accelerated in septuagenarians [37, 38] and/or that the rate of loss of muscle mass before age 70 years only starts beyond e.g. the age of 45 [39], thus halving the period of atrophy between the 20s and 70s.

Some studies report that the age-related loss of muscle mass is larger in men than in women [39–41], while others show similar losses for both genders [42]. Part of the discrepancy may be due to the way changes in muscle mass are reported. In absolute terms, men lose more mass than women because men have a larger muscle mass to start with, but in percentage terms the decrease is similar for men and women, as we observed in the present 5-year longitudinal study. In line with this, we found that while baseline muscle mass was, if anything, associated with a larger loss of muscle mass, it did not correlate with the percentage age-related decline in muscle mass. It has been reported that a lower muscle mass is associated with functional impairment and physical disability [42]. Though in absolute terms, as there was no difference in the relative rate, the decrease in muscle mass occurs at a faster rate in those with larger muscles, they will reach the disability threshold later, illustrating that it is in the long run beneficial to have a larger muscle mass [43, 44].

Previous cross-sectional studies have shown that increased levels of adipose tissue may accelerate the age-related loss of muscle mass and strength in both men and women [19, 45, 46]. In the present study, the percentage muscle loss over 5 years was positively related to the percentage body fat in men, but this was not the case for women. Particularly visceral fat mass is an important source of inflammatory cytokines [47] and an increase in the fat mass is likely to contribute to chronic low-grade systemic inflammation in older people that can cause muscle wasting and dysfunction [48]. These observations stress the benefit of a low body fat percentage for skeletal muscle health in old age and hence the importance of a healthy diet and regular physical activity [36, 49].

Previously, we observed in a cross-sectional study that the quadriceps muscles were 30% and the other muscles in the thigh only 18% smaller in older people in their 70s than young-adults in their 20s [15]. Here, we did not see a differential rate in loss of muscle mass over the 5-year period, suggesting that all muscles in the thigh atrophy at the same rate.

### Limitations

One limitation of the study is the relatively small sample size. However, it appeared that the population of the present study was a representative sample of the larger population in our previous study [15]. The participants were relatively healthy and were living independently at baseline and follow-up; it is likely that our results do not translate to those losing independence in later life.

## Conclusion

Both DXA and MRI showed a similar percentage decline over a 5-year period in septuagenarians. The decline was independent of the muscle mass at baseline and similar for men and women. A high percentage body fat was associated with a faster rate of muscle decline in men. These data indicate that (1) DXA can be used to assess longitudinal changes in muscle mass in older people, (2) longitudinal studies of septuagenarians reveal a greater rate of muscle decline than cross-sectional comparisons of young and older adults, (3) a low muscle mass is not indicative of a higher rate of age-related muscle declines and (4) increased body fatness was associated with a greater rate of age-related muscle loss in men.
